# An Efficient Algorithm for Some Highly Nonlinear Fractional PDEs in Mathematical Physics

**DOI:** 10.1371/journal.pone.0109127

**Published:** 2014-12-19

**Authors:** Jamshad Ahmad, Syed Tauseef Mohyud-Din

**Affiliations:** Department of Mathematics, Faculty of Sciences, HITEC University, Taxila, Pakistan; National Institute of Genomic Medicine, Mexico

## Abstract

In this paper, a fractional complex transform (FCT) is used to convert the given fractional partial differential equations (FPDEs) into corresponding partial differential equations (PDEs) and subsequently Reduced Differential Transform Method (RDTM) is applied on the transformed system of linear and nonlinear time-fractional PDEs. The results so obtained are re-stated by making use of inverse transformation which yields it in terms of original variables. It is observed that the proposed algorithm is highly efficient and appropriate for fractional PDEs and hence can be extended to other complex problems of diversified nonlinear nature.

## Introduction

Fractional differential equations arise in almost all areas of physics, applied and engineering sciences [1]–[8]. In order to better understand these physical phenomena as well as further apply these physical phenomena in practical scientific research, it is important to find their exact solutions. The investigation of exact solution of these equations is interesting and important. In the past several decades, many authors mainly had paid attention to study the solution of such equations by using various developed methods. Recently, the variational iteration method (VIM) [1]–[3] has been applied to handle various kinds of nonlinear problems, for example, fractional differential equations [4], nonlinear differential equations [5], nonlinear thermo elasticity [6], nonlinear wave equations [7]. In Refs. [8]–[13] Adomian's decomposition method (ADM), homotopy perturbation method (HPM), homotopy analysis method (HAM) and variation of parameter method (VPM) are successfully applied to obtain the exact solution of differential equations. In the present article, we used reduced differential transform method (RDTM) [14]–[18], to construct an appropriate solution of some highly nonlinear time-fractional partial differential equations of mathematical physics.

## Preliminaries

In this section, we give some basic formula and results about fractional calculus, and then we discuss the analysis reduced differential transform method (RDTM) to fractional partial differential equations.

### 1 Jumarie's Fractional Derivative

Some useful results and properties of Jumarie's fractional derivative were summarized [20]. 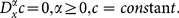
(1)

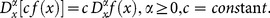
(2)

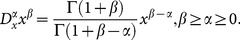
(3)

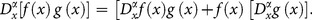
(4)

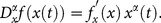
(5)


### 2 Fractional Complex Transform

The fractional complex transform was first proposed [19] and is defined as 
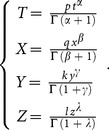
(6)where *p*, *q*, *k*, and *l* are unknown constants, 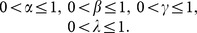


### 3 Reduced Differential Transform Method (RDTM)

To demonstrate the basic idea of the DTM, differential transform of 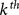 derivative of a function 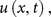 which is analytic and differentiated continuously in the domain of interest, is defined as
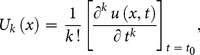
(7)


The differential inverse transform of 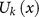 is defined as follow
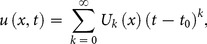
(8)


Eq. (8) is known as the Taylor series expansion of 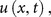
around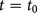. Combining Eq. (7) and (8)
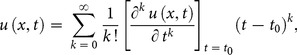
(9)when 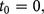
above equation reduces to
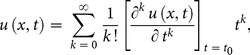
(10)and Eq. (2) reduces to



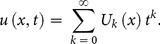
(11)


**Theorem 1:** If the original function is 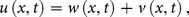 then the transformed function is 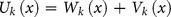



**Theorem 2:** If 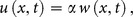
then 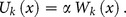


**Theorem 3:** If 
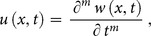
then 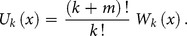


**Theorem 4:** If 
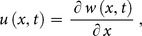
then 
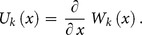


**Theorem 5:** If 
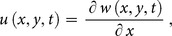
then 
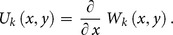


**Theorem 6:** If 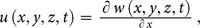
then 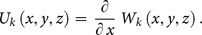


**Theorem 7:** If 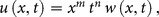
then 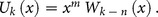


**Theorem 8:** If 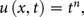
then 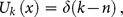
where 
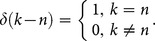


**Theorem 9:** If 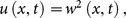
then 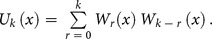


### 4 Numerical Applications of RDTM

In this section, we shall apply the reduced differential transform method (RDTM) to construct approximate solutions for some nonlinear fractional PDEs in mathematical physics and then compare approximate solutions to the exact solutions as follows.

#### 4.1 Fornberg-Whitham (FW) Equation [21]



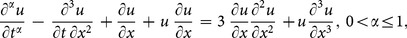
(12)with the initial conditions



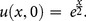
(13)Applying the transformation [19], we get the following partial differential equation
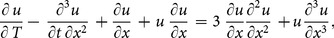
(14)


Applying the differential transform to Eq. (14) and Eq. (13), we obtain the following recursive formula 
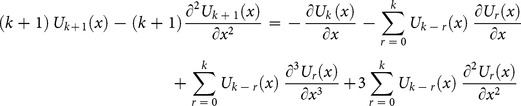
(15)using the initial condition, we have



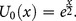
(16)Substituting Eq. (16) into (15), we obtain the following values of 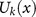 successively, 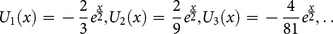



The series solution is given by
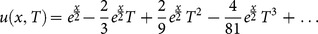



The inverse transformation will yields
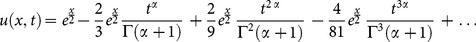
(17)


This solution is convergent to the exact solution [22]

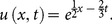
(18)


**Figure 1:**
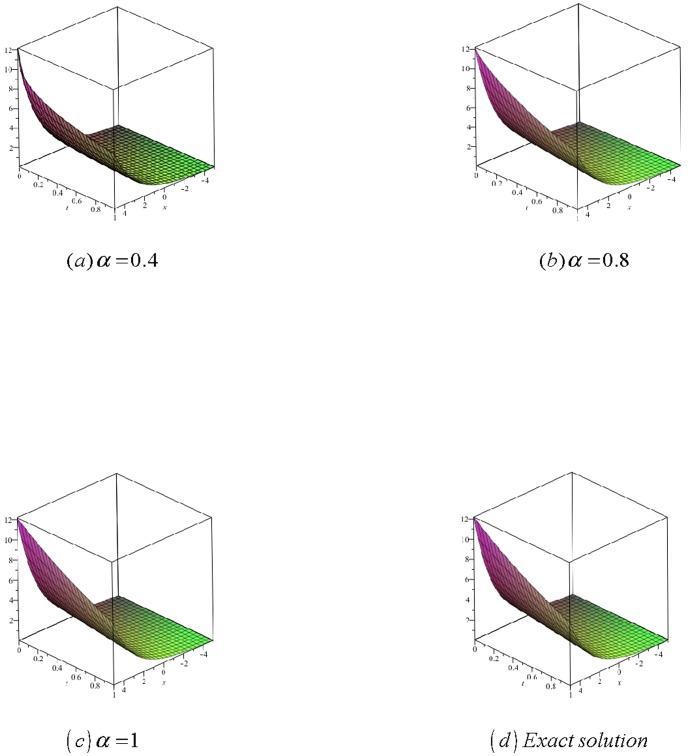
Surface plot of approximate and exact solutions of (12) for different values of *α*, using only 3rd order of RDTM solution.

**Fig. 1** **(a–d)** **:** Surface plot of approximate and exact solutions of (12) for different values of 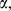 using only 3^th^ order of RDTM solution are:

#### 4.2 Modified Fornberg-Whitham (MFW) Equation [23]



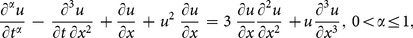
(19)with the initial conditions



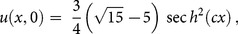
(20)where 
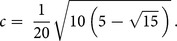


Applying the transformation [19], we get the following partial differential equation
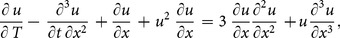
(21)


Applying the differential transform to Eq. (21) and Eq. (20), we obtain the following recursive formula 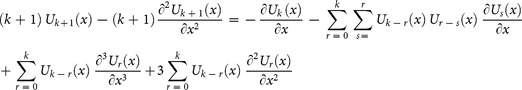
(22)using the initial condition, we have



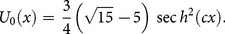
(23)Now, substituting Eq. (21) into (20), we obtain the following values 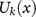 successively, 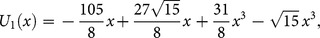


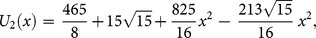


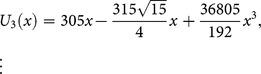



Finally, after applying the inverse transformation the approximate solution is
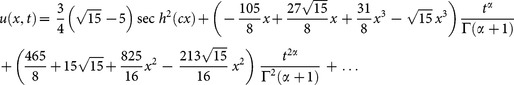
(24)


The exact solution [23] of this problem is
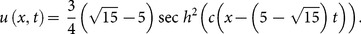
(25)


**Figure 2:**
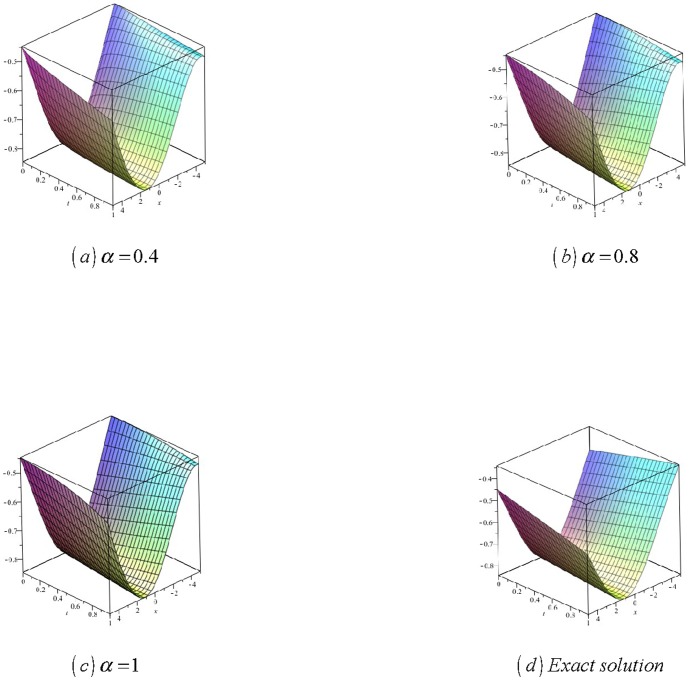
Surface plot of approximate and exact solutions of (19) for different values of *α*, using only 3rd order of RDTM solution.

**Fig. 2** **(a–d)** **:** Surface plot of approximate and exact solutions of (19) for different values of 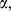 using only 3^th^ order of RDTM solution are:

#### 4.3 Sharma-Tasso-Olver (STO) Equation [24]



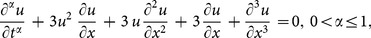
(26)with the initial conditions



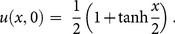
(27)Applying the transformation [19], we get the following partial differential equation
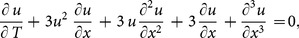
(28)


Applying the differential transform to Eq. (28) and (27), we obtain the following recursive formula 
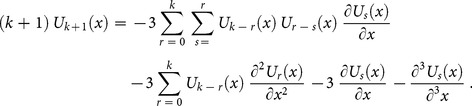
(29)using the initial condition, we have



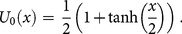
(30)Now, substituting Eq. (30) into (29), we obtain the following values 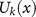 successively, 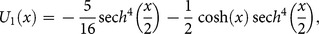


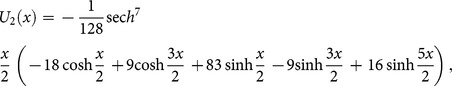


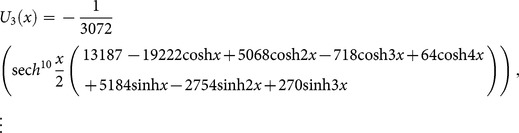



The series solution is given by
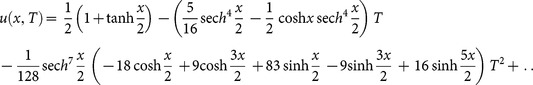



Finally, the inverse transformation will yields the solution
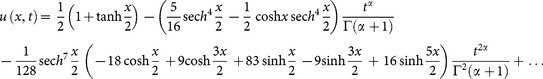
(31)


Where the exact solution is
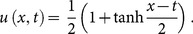
(32)


**Figure 3:**
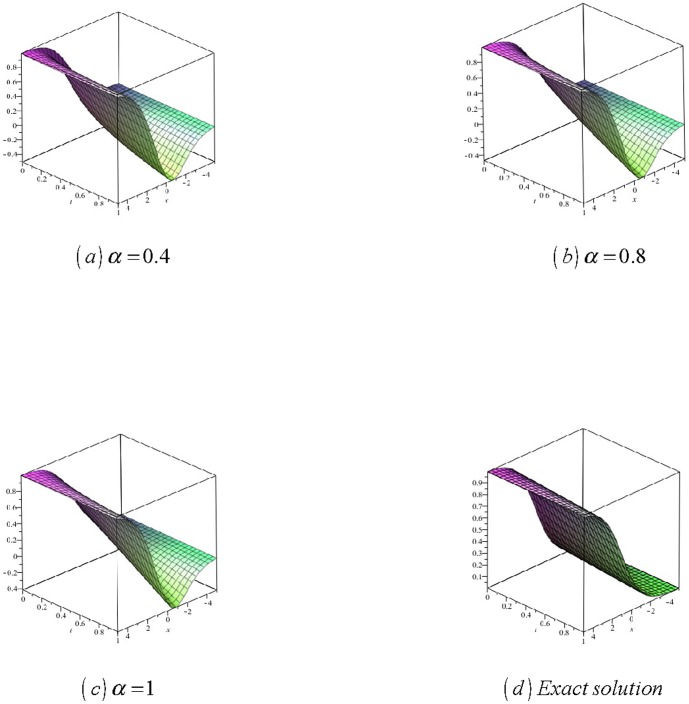
Surface plot of approximate and exact solutions of (26) for different values of *α*, using only 3rd order of RDTM solution.

**Fig. 3** **(a–d)** **:** Surface plot of approximate and exact solutions of (26) for different values of 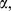 using only 3^th^ order of RDTM solution are:

#### 4.4 Gardner Equation [25]



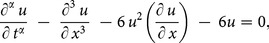
(33)with the initial condition



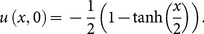
(34)Applying the transformation [19], we get the following partial differential equation
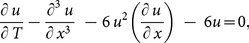
(35)


Applying the RDTM to (35) and (34), we obtain the recursive relation
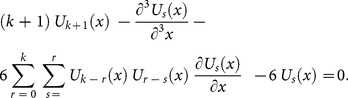
(36)using the initial condition, we have



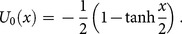
(37)Substituting Eq. (37) into Eq. (36), we obtain the following values 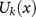 successively, 
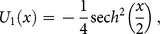


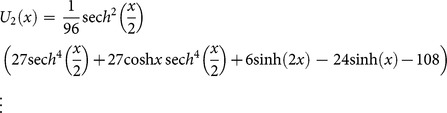



The series solution is given by
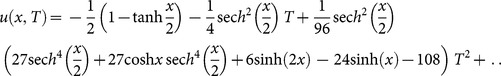



Finally, the inverse transformation will yields the solution
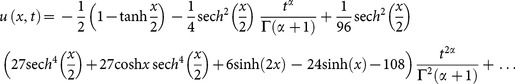
(38)


Where the exact solution is
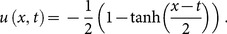
(39)


**Figure 4:**
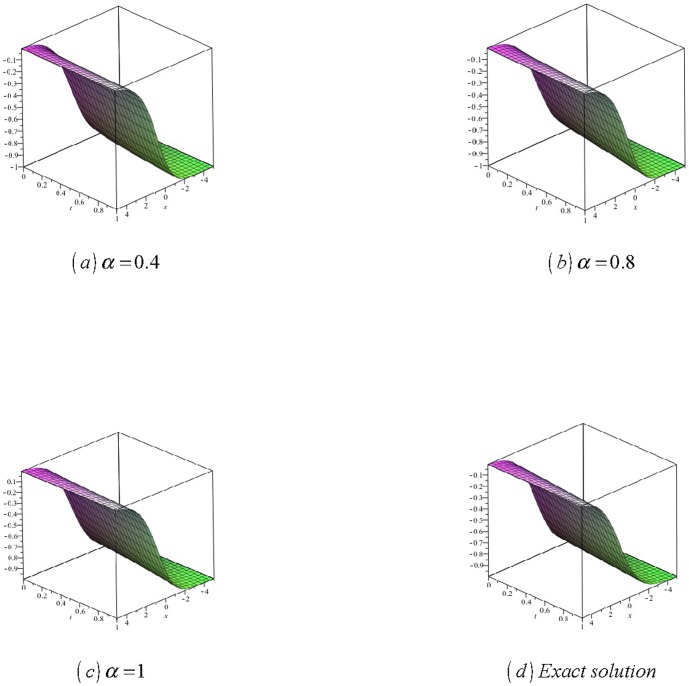
Surface plot of approximate and exact solutions of (33) for different values of *α*, using only 3rd order of RDTM solution.

**Fig. 4** **(a–d)** **:** Surface plot of approximate and exact solutions of (33) for different values of 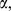 using only 3^th^ order of RDTM solution are:

#### 4.5 Variant Water Wave (VWW) equation [26]



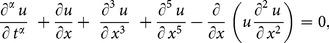
(40)with initial condition
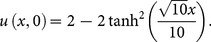
(41)


Applying the transformation [19], we get the following partial differential equation
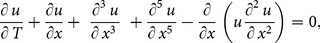
(42)


Applying the RDTM to (42) and (41), we obtain the recursive relation
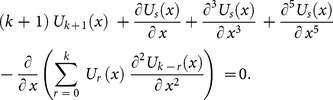
(43)using the initial condition, we have



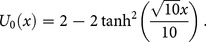
(44)Substituting Eq. (44) into (43), we obtain the following values 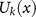 successively, 
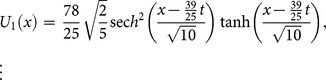



The series solution is given by
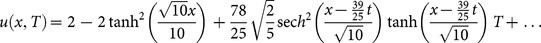



Finally, the inverse transformation will yields the solution
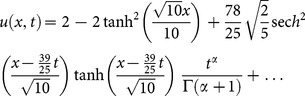
(45)


The exact solution [26] is given by
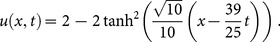
(46)


**Figure 5:**
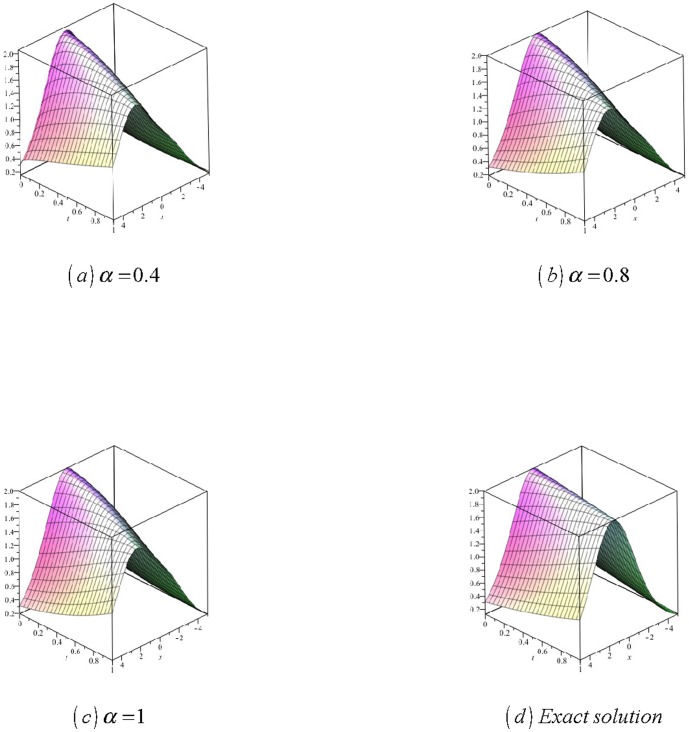
Surface plot of approximate and exact solutions of (40) for different values of *α*, using only 3rd order of RDTM solution.

**Fig. 5** **(a–d)** **:** Surface plot of approximate and exact solutions of (32) for different values of 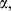 using only 3^th^ order of RDTM solution are:

## Conclusions

Applied fractional complex transform (FCT) proved very effective to convert the given fractional partial differential equations (FPDEs) into corresponding partial differential equations (PDEs) and the same is true for its subsequent effect in Reduced Differential Transform Method (RDTM) which was implemented on the transformed system of linear and nonlinear time-fractional PDEs. The solution obtained by Reduced Differential Transform Method (RDTM) is an infinite power series for appropriate initial condition, which can in turn express the exact solutions in a closed form. The results show that the Reduced Differential Transform Method (RDTM) is a powerful mathematical tool for solving partial differential equations with variable coefficients. Computational work fully re-confirms the reliability and efficacy of the proposed algorithm and hence it may be concluded that presented scheme may be applied to a wide range of physical and engineering problems.

## References

[1] Noor MA, Mohyud-Din ST (2008) Modified variational iteration method for heat and wave-like equations. Acta Appl Math 104:257–269.

[2] Abbasbandy S (2007) A new application of He's variational iteration method for quadratic Riccati differential equation by using Adomian's polynomials. J Comput Appl Math 207:59–63.

[3] Abbasbandy S (2007) Numerical solutions of nonlinear Klein-Gordon equation by variational iteration method. Internat J Numer Meth Engrg 70:876–881.

[4] He JH (1999) Some applications of nonlinear fractional differential equations and their approximations. Bull Sci Technol 15:86–90.

[5] Bildik N, Konuralp A (2006) The use of variational iteration method, differential transform method and Adomian decomposition method for solving different types of nonlinear partial differential equation. Int J Non-Linear Sci Numer Simul 7:65–70.

[6] Sweliam NH, Khader MM (2007) Variational iteration method for one dimensional nonlinear thermo-elasticity. Chaos Soliton Fract 32:145–149.

[7] Soliman AA (2006) A numerical simulation and explicit solutions of KdV-Burgers' and Lax's seventh-order KdV Equations. Chaos Solitons Fract 29:294–302.

[8] Momani S, Al-Khaled K (2005) Numerical solution for systems of fractional differential equations by the decomposition method. Appl Math Comput 162:1351–65.

[9] Odibat Z, Momani S (2007) Numerical solution of Fokker-Planck equation with space-and time-fractional derivatives. Phys Lett A 369:349–358.

[10] Yıldırım A, Koçak H (2009) Homotopy perturbation method for solving the space-time fractional advection-dispersion equation. Adv Water Resour 32:1711–1716.

[11] Matinfar M, Saeidy M (2010) Application of Homotopy Analysis method to fourth order parabolic partial differential equations. Appl Appl Math 5:70–80.

[12] Mohyud-Din ST, Noor MA, Waheed A (2009) Variation of parameter method for solving sixth-order boundary value problems. Commun Korean Math Soc 24:605–615.

[13] Mohyud-Din ST, Noor MA, Waheed A (2010) Variation of parameter method for initial and boundary value problems. World Appl Sci J 11:622–639.

[14] Jang MJ, Chen CL, Liu YC (2006) Two-dimensional differential transform for partial differential equations. Appl Math Comput 181:767–774.

[15] Arikoglu A, Ozkol I (2007) Solution of fractional differential equations by using differential transform method. Chaos Soliton Fract 34:1473–1481.

[16] Zhou JK (1986) Differential transform and its applications for Electrical Circuits. Huazhong University Press Wuhan, China.

[17] Merdan M, Gokdogan A (2011) Solution of nonlinear oscillators with fractional nonlinearities by using the modified differential transformation method. Math Comput Appl 16:761–772.

[18] Kurnaz A, Oturance G (2005) The differential transforms approximation for the system of ordinary differential equations. Int J Comput Math 82:709–719.

[19] Li ZB, He JH (2010) Fractional Complex Transform for Fractional Differential Equations. Math Comput Appl 15:970–973.

[20] Jumarie G (2006) Modified Riemann-Liouville Derivative and Fractional Taylor series of Non-differentiable Functions Further Results. Comput Math Appl 51:1367–1376.

[21] Whitham GB (1967) Variational methods and applications to water wave. Proc R Soc Lond Ser A 299:6–25.

[22] Fornberg B, Whitham GB (1978) A numerical and theoretical study of certain nonlinear wave phenomena. Philos A Trans R Soc Lond Ser A 289:373–404.

[23] He B, Meng Q, Li S (2010) Explicit peakon and solitary wave solutions for the modified Fornberg-Whitham equation. Appl Math Comput 217:1976–1982.

[24] Olver PJ (1977) Evolution equations possessing infinitely many symmetries. Int J Math Phys 18:1212–1215.

[25] Wazwaz AM (2007) New solitons and kink solutions for the Gardner equation. Comm Nonlin Sci Numer Simul 12:1395–404.

[26] Rawashdeh M (2013) Improved approximate solutions for nonlinear evolutions equations in mathematical physics using the deduced differential transform method. j Appl Math Bioinfom 3:1–14.

